# Supplementary material for: Sonic Hedgehog-Gli1 Signaling and Cellular Retinoic Acid Binding Protein 1 Gene Regulation in Motor Neuron Differentiation and Diseases
Source: Int J Mol Sci. 2020 Jun 9;21(11):4125. doi: 10.3390/ijms21114125 (PMC7312406; doi:10.3390/ijms21114125)
Supplement: Supplementary file 1 [file ijms-21-04125-s001.pdf]

International Journal of *Molecular Sciences*

MDPI

Article

Sonic hedgehog-Gli1 signaling and cellular retinoic acid binding protein 1 gene regulation in motor neuron differentiation and diseases

Yu-Lung Lin[1#], Yi-Wei Lin[1#], Jennifer Nhieu[1#], Xiaoyin Zhang[1], and Li-Na Wei[1*]

[1]Department of Pharmacology, University of Minnesota, Minneapolis, MN, 55455, USA; yllin@umn.edu (YL.L.); linxx637@umn.edu (YW.L.); nhieu001@umn.edu (J.N.); zhan6240@umn.edu (X.Z.)

* Correspondence: weixx009@umn.edu (LN.W.); Tel: +1-612-6259402

[#] Contributed equally

Supplementary data

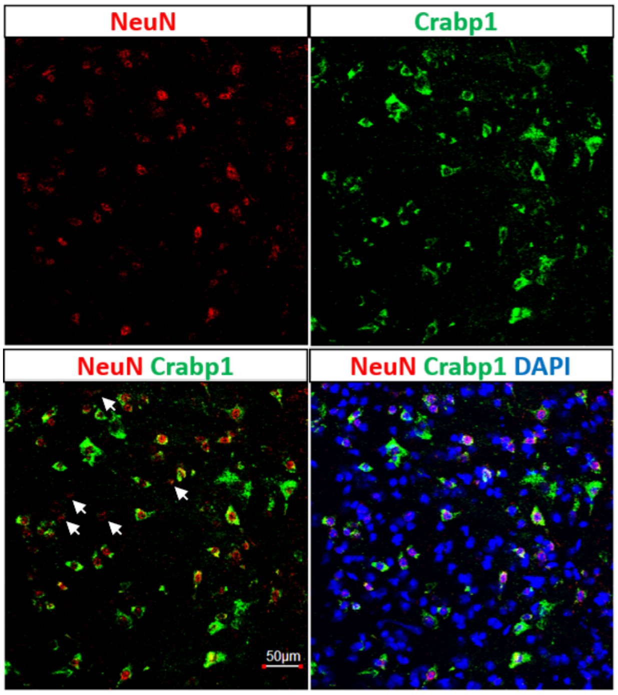

Supplementary Figure 1. Crabp1 expression in spinal neurons. Confocal microscopy images showing signals of CRABP1 (Green), general neuronal marker NeuN (Red), and cell nuclei (DAPI). White arrows mark NeuN+/Crabp1- cells, indicating subsets of Crabp1-negative spinal neurons. Scale bar = 50 um.

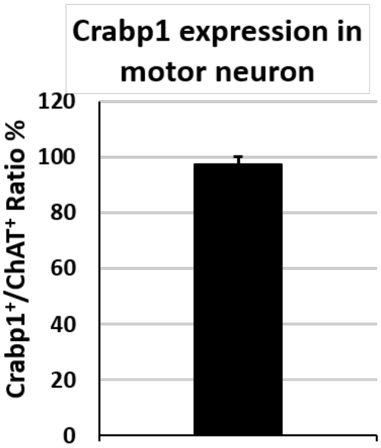

Supplementary Figure 2. Quantification of Crabp1-positive spinal motor neurons. Data of Fig. 1b, from multiple sections, are quantified to determine the ratio of Crabp1+/ChAT+ cells in the lumbar spinal section, N = 4. The result is presented as means ± SEM. The result shows that greater than 95% of spinal motor neurons are Crabp1-positive.
